# Comparison of commercially available whole-genome sequencing kits for variant detection in circulating cell-free DNA

**DOI:** 10.1038/s41598-020-63102-8

**Published:** 2020-04-10

**Authors:** Florence Mauger, Caroline Horgues, Morgane Pierre-Jean, Nouara Oussada, Lilia Mesrob, Jean-François Deleuze

**Affiliations:** 1 Université Paris-Saclay, CEA, Centre National de Recherche en Génomique Humaine, 91057 Evry, France; 2 INSERM, Université Paris Sorbonne, Paris, France; 3 Centre d’Etude du Polymorphisme Humain, Fondation Jean Dausset, Paris, France; 4 Centre de Référence, d’Innovation, d’expertise et de transfert (CREFIX), Evry, France

**Keywords:** Biological techniques, Biotechnology, Cancer, Biomarkers

## Abstract

Circulating cell-free DNA (ccfDNA) has great potential for non-invasive diagnosis, prognosis and monitoring treatment of disease. However, a sensitive and specific whole-genome sequencing (WGS) method is required to identify novel genetic variations (i.e., SNVs, CNVs and INDELS) on ccfDNA that can be used as clinical biomarkers. In this article, five WGS methods were compared: ThruPLEX Plasma-seq, QIAseq cfDNA All-in-One, NEXTFLEX Cell Free DNA-seq, Accel-NGS 2 S PCR FREE DNA and Accel-NGS 2 S PLUS DNA. The Accel PCR-free kit did not produce enough material for sequencing. The other kits had significant common number of SNVs, INDELs and CNVs and showed similar results for SNVs and CNVs. The detection of variants and genomic signatures depends more upon the type of plasma sample rather than the WGS method used. Accel detected several variants not observed by the other kits. ThruPLEX seemed to identify more low-abundant SNVs and SNV signatures were similar to signatures observed with the QIAseq kit. Accel and NEXTFLEX had similar CNV and SNV signatures. These results demonstrate the importance of establishing a standardized workflow for identifying non-invasive candidate biomarkers. Moreover, the combination of variants discovered in ccfDNA using WGS has the potential to identify enrichment pathways, while the analysis of signatures could identify new subgroups of patients.

## Introduction

The analysis of circulating cell-free DNA (ccfDNA) from plasma bears great promise for diagnosis, prognosis and monitoring the treatment of cancer^1^. In the context of precision medicine, the identification of novel non-invasive biomarkers is crucial but the analysis of ccfDNA is still a challenge.

Indeed, ccfDNA is low concentrated, highly fragmented and the abundance depends on the type and the stage of cancer and the pre-analytical steps^2,3–5^. Due to its properties, a complete workflow for sample preparation, library preparation, sequencing and data analysis should be performed to ensure standardization of sample analysis especially in the case of clinical cohorts^4,6,7^. Pre-analytical steps including sample collection, storage, processing and extraction were compared to maximize the yield and size of ccfDNA^3,5,8–12^. Furthermore, size analysis and quantification methods were used to evaluate the extracted ccfDNA. Sensitive approaches such as quantitative PCR, digital PCR, mass spectrometry and next generation sequencing (NGS) are commonly applied to analyze extracted ccfDNA^2^.

With the improvement of NGS analysis, whole-genome sequencing (WGS) is a great approach to identify all types of genomic alteration including single nucleotide variant (SNV), insertion and deletion (INDEL), copy number variation (CNV) and structural variant (SV) for the identification of candidate biomarkers in cancer^13^. In particular, several specific and sensitive low-coverage sequencing approaches have been applied for the analysis of CNVs from cancer plasma samples^14–20^. In addition, recent WGS studies allowed the analysis of nucleosome positioning, tumor fraction, fragmentation patterns and chromosomal and microsatellite instability using specific ccfDNA WGS methods^21–28^.

In the present work, we compared commercially available WGS kits based on Illumina sequencing for the analysis of ccfDNA. To ensure optimal analysis of samples, a sample preparation workflow was established^8^. Then, five commercially available WGS kits including one PCR-free kit and four kits based on final amplification were compared for the detection of germline and somatic mutations as well as CNVs.

## Results

Five commercially available WGS kits were compared: ThruPLEX, QIAseq, NEXTFLEX, Accel with PCR and Accel PCR-free. Each library was prepared starting with 5–10 ng of input material to obtain sufficient amount of library to sequence at 10X or 30X sequencing coverage. Both germline and somatic mutations were detected using the GATK tool and CNVs were detected using the ichorCNA tool^29–31^.

### Sample preparation

A complete workflow was developed to maximize the yield of ccfDNA extracted from plasma, based upon previously compared ccfDNA extraction methods^8^. Commercially available plasma containing K2-EDTA as an anticoagulant was chosen to optimize ccfDNA analysis^32^. Thawed plasma samples were centrifuged to remove potential contamination of high molecular weight (HMW) DNA before extraction^33^. The extractions were performed using the commonly used QIAamp Circulating Nucleic Acid kit starting with 1 mL of plasma and using 100 µL of elution volume. ccfDNA was then quantified using Fluorometric assay and the fragment length sizes were analysed by electrophoresis to normalize each sample.

A plasma control sample (HD816) was used to check the extraction efficiency and the recovery of this control sample was 80.7% +/− 4.3%. The average concentration of all extracted ccfDNA samples was 26.7+/− 13.5 ng/mL of plasma. The average fragmented size of all ccfDNA samples was 167 bp +/− 4 bp (Supplementary Fig. S1). The fragment size analysis of breast cancer 1 sample also showed HMW DNA at about 10,000 bp and the pool of healthy donors also had a peak at about 8,500 bp. Only the prostate cancer patient provided enough ccfDNA (52 ng/mL of plasma) to perform the evaluation of all library constructions. The other ccfDNA samples were analyzed using the ThruPLEX method that has been used in several other studies^15,22,23,26^.

Three fragmented control DNAs (NA12878, HD780 and HD786) were used to mimic ccfDNA and to evaluate the detected variants.

### Sequencing of library preparation

**Figure 1:**
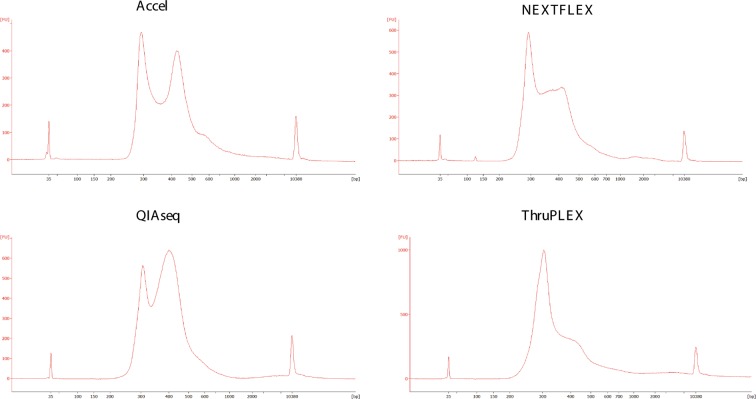
Size profiles of WGS using Accel, NEXTFLEX, QIAseq and ThruPLEX from prostate cancer plasma.

To ensure fair evaluation of the library preparation kits, a process was established starting with 5–10 ng of input material. To avoid adapter dimers, adapters were diluted for the QIAseq and NEXTFLEX protocols, PCR libraries were purified at 0.8X for QIAseq.^34^. Indeed, high ratio of adapter dimers into the library construction generates several clusters on the flow cell and consequently could reduce the sequencing capacity of the sample^34,35^. Although the adapter primer was diluted, it was still detected in the NEXTFLEX library preparation but it represented about only 1% of all clusters of this sample (Fig. 1).

The PCR-free product of Accel was not detected and consequently this protocol cannot be compared in this manuscript. For the four other library preparation kits, the number of PCR cycles was determined using qPCR assays for each sample to maximize the PCR library yield for 10X or 30X sequencing starting with this low amount of input material (5–10 ng)^36,37^. The number of PCR cycles was between 7 to 10 for all kits which correspond to the manufacturer’s recommendation except for Accel which was greater (7 instead of 2 cycles) starting with this input. Consequently optimizing the number of cycles provides enough quantity of library to sequence at either 10X or 30X.

Finally, PCR libraries were then quantified by qPCR and each size of library is analysed for equimolar pooling of samples (Fig. 1).

The four library preparation kits were sequenced at 10X and/or 30X coverage. The median coverage and percentage of paired-end reads (PE) of all 30 WGS samples are shown in Supplementary Fig. S2. Although, the median coverage is similar for 10X or 30X sequencing, Accel kit shows the highest median coverage. The percentages of PE reads are not significantly different between all kits (p-values between 0.19 and 0.75). Furthermore, the ThruPLEX library constructions from plasma samples show that the median coverage (10.3X+/− 2.5X), the percentage of PE reads (90.4% +/−6.8%) and the insert size (163 bp + /− 14.6 bp) depend also on the type of plasma sample. Finally, for the library construction of NEXTFLEX, 5 ng of starting material was used as recommended by the manufacturer except for the NA12878 WGS at 30X. WGS comparison of all NA12878 samples at 30X shows that 10 ng of starting input can also be used for NEXTFLEX.

### Detection of targeted variants from the reference control sample

To compare the sensitivity and specificity for germline and somatic mutation detection, three standard reference samples including NA12878 (Table 1), HD786 and HD780 ccfDNA reference standard were used (Table 2).

**Table 1:** Germline SNV and INDEL detection of NA12878 sample (NIST reference (HG001) of GIAB (https://www.nist.gov/programs-projects/genome-bottle) from Accel, NEXTFLEX, QIAseq and ThruPLEX kits.

WGS methods	Median coverage (X)	Number of SNV	Number of INDEL	SNV_TPR %	SNV_PPV %	INDEL_TPR %	INDEL_PPV %
Accel	12,0	3616493	702550	95,96	99,42	87,47	96,06
	38	3838215	927664	99,9	99,68	98,85	93,18
NEXTFLEX	9,0	3303878	582961	88,37	98,98	76,92	94,7
	37	3810345	882677	99,82	99,66	98,04	94,17
QIAseq	8,0	3209340	598051	85,35	98,41	74,59	89,64
	35	3808366	931168	99,77	99,62	97,22	87,22
ThruPLEX	8,0	3084349	575960	81,44	97,18	68,1	81,16
	33	3777238	916835	99,56	99,54	93,45	80,33

The number of SNVs and INDELs and the TPR and PPV of each detected.

**Table 2:** Somatic SNV detection of HD780 and HD786 samples from Accel, NEXTFLEX, QIAseq and ThruPLEX kits.

Sample	WGS methods	Median coverage (X)	Detected SNV
HD780	NEXTFLEX	9,0	
	Accel	9,0	
		45,4	
	ThruPLEX	8,0	PIK3CA (E545K)
		40,0	PIK3CA (E545K)
	QIAseq	8,0	KRAS (G12D)
HD786	NEXTFLEX	8,0	
	Accel	9,0	PIK3CA (E545K)
		47,0	PIK3CA (E545K)
	ThruPLEX	8,0	
		38,0	PIK3CA (E545K) and GNA11 (Q29L)
	QIAseq	8,0	

NA12878 DNA was used to assess whether 10X or 30X sequencing coverage was sufficient to detect the correct germline mutation (Table 1). The true positive rate (TPR) and the positive predictive value (PPV) were calculated to compare the sensitivity and the specificity of detection of known germline SNPs and INDELs in this sample (Table 1). The Accel method detects more SNVs and INDELs than the other kits and it has a higher TPR and PPV of SNVs and INDELs especially for 10X read depth. In addition, for 30X read depth, the TPR and PPV of SNVs are higher than 99.5% for each method and the TPR and PPV of INDELs is between 93.4–98.85% and 80.33–94.13% respectively. The TPR and PPV of INDELs are lower than those of SNVs because INDELs are usually more difficult to detect. Finally, the TPR of INDELs at 30X (≥93.45%) is higher than the TPR at 10X (≤87.47%) whereas the PPV of 30X(80.33% to 94.17%) is lower than the PPV of 10X(81.16% to 96.06%) for all methods.

Furthermore, WGS of the NA12878 sample using the NEXTFLEX kit was performed using 5 ng for 10X, according to the manufacturer’s recommendation, and 10 ng for 30X whereas the three other WGS kits are prepared starting with 10 ng of input material for both 10X and 30X coverage. For both coverage and input, NEXTFLEX is the second best kit for the detection of germline variants.

Moreover, HD780 control sample has six somatic SNVs at ∼5%: *EGFR* (L858R and T790M), *KRAS* (G12D), NRAS (Q61K and A59T) and *PIK3CA* (E545K) genes. HD786 contains three somatic SNVs at ∼5%: *GNA11* (Q209L) and *AKT1* (E17K) and *PIK3CA* (E545K) genes. These two references also contain two INDELs: *EGFR* gene (V769-D770insASV and Δ756-A750). Table 2 shows that both 10X and 30X read depth are unable to detect INDELs and are not sufficient to detect all somatic SNVs in the two samples. The SNV analysis of 10X of HD780 sample showed that *KRAS* (G12D) SNV was detected by the QIAseq method and *PIK3CA* (E545K) SNV was detected by the ThruPLEX method that is confirmed at 30X. For the HD786 sample, only Accel method detects *PIK3CA* (E545K) at 10X. In addition, the comparison of 30X Accel and ThruPLEX sequencing of HD786 showed that Accel detected only one somatic SNV in *PIK3CA* (E545K) whereas ThruPLEX detected two somatic SNVs: in *PIK3CA* (E545K) and in the *GNA11* genes (Q209L).

Finally, HD786 sample has also two CNVs: 4.5 copies of *MET* gene chromosome 7 and 9.5 copies of *MYCN* gene chromosome 2. Both targeted CNVs were detected using high amplification calling criteria for all kits in 10X WGS of HD786.

### Comparison of WGS methods

The four library preparation kits were compared using the same control samples and prostate plasma sample for the detection of different type of variants (SNVs, INDELs, and CNVs).

**Figure 2:**
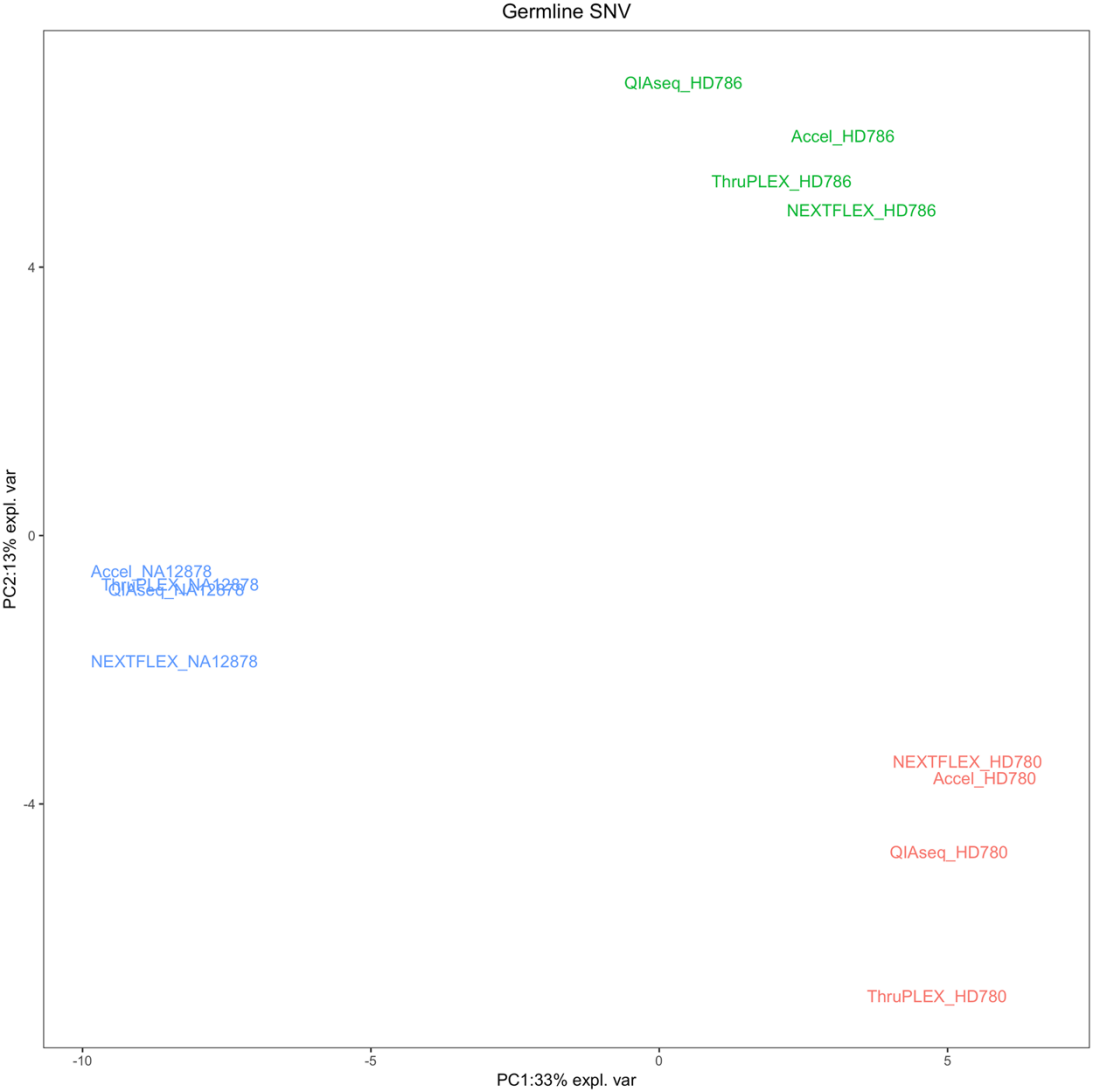
Principal component analysis of germline SNV for QIAseq, Accel, NEXTFLEX and ThruPLEX WGS of 10X of the NA12878, HD780 and HD786 samples. NA12878 sample is blue, HD786 sample is green and HD780 sample is red.

The principal component analysis (PCA) of 10X WGS of NA12878, HD780 and HD786 samples for the detection of germline SNVs showed that the two first principal components captured 46% of the variation (Fig. 2). The first component of PCA captured 33% of the total variance of the samples and showed an evident split between the two plasma control samples (HD780 and HD786) and NA12878 sample. The second component that explained 13% of the total variance, split the three samples and also highlights the variability of the four kits that depends on each sample.

Furthermore, Accel and ThruPLEX WGS were also compared for the detection of germline SNVs at 10X and 30X coverage from these three same samples (Supplementary Fig. S3). The two first principal components captured 58% of the variation and NA12878, HD780 and HD786 samples were separated. Although, WGS results from both kits of the same sample are similar, the WGS variability of kits and coverage depends on each sample.

**Figure 3:**
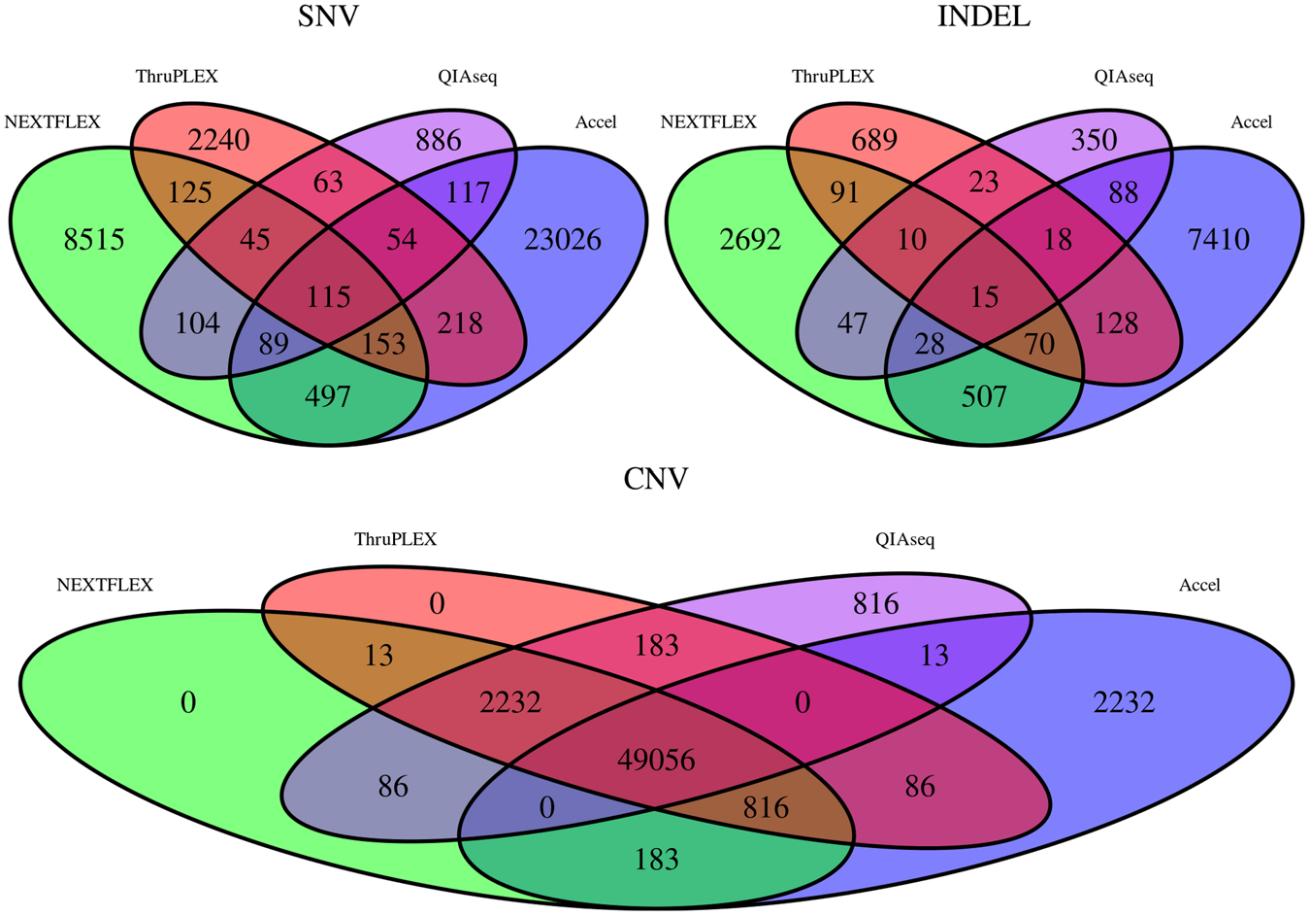
Venn diagram of the detection of somatic SNVs, INDELs and CNVs for four WGS methods from the same prostate cancer plasma sample. NEXTFLEX method is green, ThruPLEX method is red, QIAseq method is purple and Accel method is blue.

In addition, detection of SNVs, INDELs and CNVs from all kits were compared on the same prostate cancer plasma sample. Only a few somatic mutations (115 SNVs and 15 INDELs) were detected by all four WGS methods whereas most of the CNVs were detected by all four WGS methods (Fig. 3). Furthermore, for each type of variant, the Accel method uncovers more unique variants then the other methods. The biggest number of common SNVs and INDELs are between Accel and NEXTFLEX and the proportion of common CNVs is bigger between ThruPLEX, QIAseq and NEXTFLEX.

Although unique variants were detected for each kit, Table 3 showed that all kits had a significantly high proportion of common germline SNVs. Accel had a significantly small number of common germline INDELs compared to the other kits whereas ThruPLEX, NEXTFLEX and QIAseq had a significantly high proportion of common germline INDELs combined.

**Table 3:** Adjusted p-values for under representation and over-representation tests of the variant detection of NEXTFLEX, Accel, ThruPLEX and QIAseq from the prostate cancer plasma sample.

Kits	Adjusted p-values for under-representation test					Adjusted p-values for over-representation test				
	CNV	Germline INDEL	Somatic INDEL	Germline SNV	Somatic SNV	CNV	Germline INDEL	Somatic INDEL	Germline SNV	Somatic SNV
Accel and QIAseq	0	0	0	1	0	1	1	1	0	1
Accel and ThruPLEX	1	0	0	1	0	0	1	1	0	1
NEXTFLEX and Accel	1	0	0	1	0	0	1	1	0	1
NEXTFLEX and QIAseq	1	1	0	1	0.168	0	0	1	0	1
NEXTFLEX and ThruPLEX	1	1	0	1	0	0	0	1	0	1
ThruPLEX and QIAseq	1	1	1	1	1	0	0	0.103	0	0

Furthermore, Accel had a significantly low proportion of common somatic SNVs compared to the other kits and also NEXTFLEX with ThruPLEX. ThruPLEX and QIAseq had a significantly high proportion of common somatic SNVs. Except for ThruPLEX and QIAseq, the proportion of common somatic INDELs between kits was significantly high. Finally, for Accel and QIAseq, the proportion of common CNVs was significantly low and other kits had a significantly large number of common CNVs.

### Analysis of plasma samples

**Figure 4:**
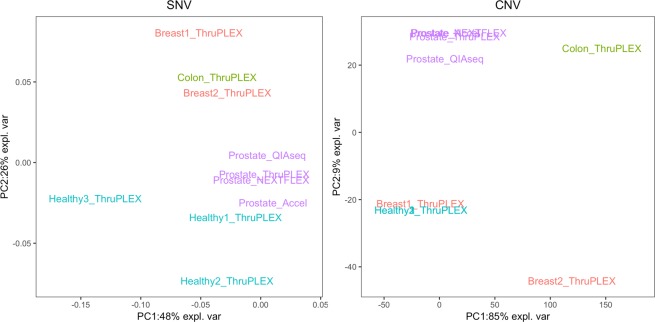
Principal component analysis of somatic SNV and CNV detection of ccfDNA samples from 10X coverage. The ccfDNA samples are: purple for prostate cancer from NEXTFLEX, ThruPLEX, QIAseq and Accel. The ccfDNA from ThruPLEX are blue for healthy 1, healthy 2 and healthy 3 samples, red for breast cancer 1 and breast cancer 2 samples and green for colon cancer patient.

The detection of somatic SNVs and CNVs of all plasma samples were compared in Fig. 4. Both PCA for SNV and CNV detection show the diversity of the ccfDNA samples analyzed by ThruPLEX kits. For CNV detection, Accel, ThruPLEX and NEXTFLEX cluster together whereas QIAseq is close to the three other kits in the same prostate cancer plasma sample. For SNV calling, ThruPLEX and NEXTFLEX are grouped together whereas the QIAseq and the Accel are similar. The detection of CNVs in the three healthy plasma samples and Breast cancer 1 plasma are grouped together which could be explained by the presence of HMW DNA in the Breast cancer 1 ccfDNA (Supplementary Fig. S1).

**Figure 5:**
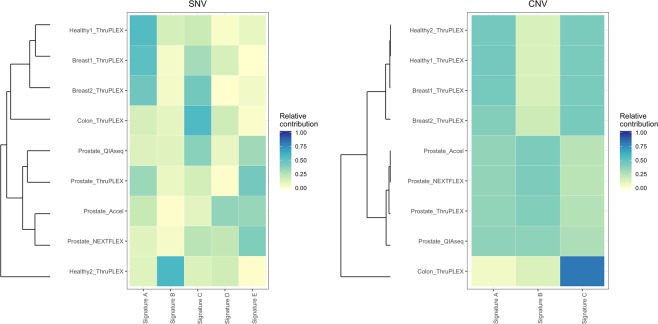
Heatmaps of SNV and CNV signatures of ccfDNA samples.

In addition, SNV and CNV signature analysis of these samples showed that prostate samples share similar signatures while the other types of plasma samples had different ones (Fig. 5). The breast cancer 1 sample, which contains HMW DNA, has similar CNV signatures with the two healthy individual samples whereas the female healthy individual 1 and breast cancer 1 have similar SNV signatures. Although the signatures of the four kits clustered, Accel and NEXTFLEX were more alike. ThruPLEX were similar CNV patterns with the two other kits and ThruPLEX and QIAseq were alike for SNV signatures.

Finally, pathway enrichment analysis was performed using common detected genes of the four kits (Supplementary Table S1) of the prostate cancer plasma individual. The most significant p-values (between 1.7 × 10^−16^ to 4.9 × 10^−8^) were obtained with the Spliceosome, Ubiquitin mediated proteolysis, NF-kappa B signalling and Ribosome pathways, all of which are known to be involved in prostate cancer^38–41^.

## Discussion

In the current manuscript, we compared five commercially available WGS kits to identify novel candidate biomarkers from ccfDNA.

The comparison of one PCR-free method versus four kits based upon final amplification before WGS showed that the four PCR based kits could be used starting with less ccfDNA. NEXTFLEX produced similar results to the other kits starting with 5 ng of input instead of 10 ng used for the other kits (Tables 1 and 2). All WGS results were similar in terms of coverage, and they are not significantly different in terms of the percentage of PE reads (Supplementary Fig. S2). Samples are more different for SNV and CNV detection than the individual difference between kits (Figs. 2, 4 and 5 and Supplementary Fig. S3). Although, there are variabilities between kits and/or coverage which depend on the sample, all kits have significant common numbers of SNVs, CNVs and similar CNV and SNV signatures (Tables 1 and 3 Figs. 2, 4 and 5 and Supplementary Fig. S3). They allowed the detection of the two targeted CNVs from the control ccfDNA using 10X or 30X sequencing. Indeed, in these studies, low or ultra-low coverage WGS allowed the detection of CNVs from ccfDNA^14,16,23^. Accel had the best coverage and detected more variants than the other kits including several unique SNVs, INDELs and CNVs (Tables 1 and 3, Fig. 3 and Supplementary Fig. S2). This detection of unique variants could be explained by the use of more PCR cycles compared to the manufacturer’s protocol for Accel. Accel and NEXTFLEX were alike for SNV and CNV patterns and ThruPLEX and QIAseq were alike for SNV signatures (Fig. 5). The overlap of somatic SNVs detected by NEXFLEX and QIAseq is not significantly high or low and only ThruPLEX and QIAseq have a significant high level of common somatic SNV (Table 3). For the detection of low-abundant (5%) SNVs, only a few somatic SNVs were detected for all kits (Table 2). It could be due to the low-coverage but ThruPLEX allows the detection of more low-abundant somatic mutations than the other kits. In addition, the ThruPLEX kit enabled the analysis of CNV, microsatellite instability, nucleosome footprint and fragment size analysis in ccfDNA^15,22,23,26,27^. In our article, we showed that the ThruPLEX kit allowed the analysis of variants from various types of plasma samples (Figs. 4 and 5).

Due to the lack of standardized processing for ccfDNA sample preparation, a pre-analytical workflow should be developed for collection, storage, pre-processing, extraction and quantification of extracted ccfDNA^3,4,6,7^. In our study, we developed a workflow based on our previous comparison of extraction methods for plasma with EDTA anticoagulant but it could also be used for blood collection tubes with an improvement of the pre-processing step^8^. This sample preparation workflow allowed the extraction of 1 mL of plasma using the most commonly used QIAgen Nucleic acid kit with an elution volume of 100 µL to maximize the recovery of extracted ccfDNA from plasma. In addition, other liquid biopsies such as serum, cerebrospinal fluid or urine could be performed by further optimization of the protocol. Furthermore, ccfDNA samples are quantified using this assay or a qPCR of *KPN* sequences for samples which are below the limit of detection of the fluorometric assay^8,42^. Furthermore, fragment size analysis is performed before sequencing to verify the size of ccfDNA and to ensure it does not contain HMW DNA which is about 10,000 bp (Supplementary Fig. S1)^43^. The HMW DNA could be removed using purification beads^43^. Breast cancer 1 ccfDNA contains HMW DNA and it seems to be similar to healthy ccfDNA samples for the detection of CNVs and both CNV and SNV signatures for female healthy individual 1 (Figs. 4 and 5 and Supplementary Fig. S1). The average CNV of breast 1 cancer could be reduced to two copies due to the presence of HMW DNA as expected in healthy individual plasma. Finally, specific and sensitive approaches to characterize extracted ccfDNA should be performed to minimise the quantity of ccfDNA used for quality control.

For the comparison of the five methods, we have also developed a process starting with a 5 or 10 ng of ccfDNA that involved determination of a number of PCR cycles for each library construction, quantification and size analysis of WGS to have optimal pooling and enough starting material for 10X and 30X sequencing (Fig. 1). We have modified the adapter conditions for NEXTFLEX and for QIAseq to avoid adapter dimers because a high ratio of adapter dimers in the library could reduce the sequencing efficiency of the sample^34,35^. For the PCR based library preparation, the number of PCR cycle was previously determined using qPCR assays for each sample to have enough PCR product to be sequenced^1,2^. Indeed, high number of PCR cycling could result in more PCR-induced variations. We did not obtain enough library preparation with the Accel PCR-free protocol for sequencing. By increasing the quantity of ccfDNA with higher volume of plasma, this PCR-free protocol could be used. Consequently, this protocol has not been compared with the four other protocols but the Accel protocol with final amplification has been obtained (Fig. 1). Finally, the number of PCR cycles is decreased by starting with higher quantities of ccDNA.

We performed variant calling using a commonly used GATK pipeline and CNV analysis using the ichorCNA tool, which was applied for ccfDNA analysis^13,24,31^. Tables 1 and 2 show that the INDEL calling is less effective than SNV calling for all the compared kits and for both germline mutation and somatic mutation detection. The intersection of somatic INDELs identified by ThruPLEX and QIAseq is not significantly high or low (Table 3). The INDEL calling using the GATK tool is less efficient; other INDEL calling tools can be used to enhance the INDEL detection of WGS data^44^. In addition, 10X and 30X coverage of the 4 WGS kits seem to be insufficient to detect all somatic mutations with 5% abundance (Table 2). Increasing the coverage could improve the detection of low-abundant somatic mutations. Besides, several algorithms have been recently developed and they could be used to analyse WGS data of ccfDNA samples to improve the detection of somatic variants in the context of precision medicine^13,45–47^.

Finally, although the 10X coverage was not optimal to detect low-abundant mutations, we showed that the combination of detection of common SNVs, INDELs and CNVs of prostate cancer plasma from four kits has the potential to identify pathways for a given disease (Supplementary Table S1). More particularly, analysis of omic data from ccfDNA could improve knowledge in cancer^48^. Furthermore, the analysis of SNV or CNV patterns could identify clusters of patients for the discovery of new disease subgroups (Fig. 5). A complete workflow for liquid biopsy including sample preparation, library construction for low input, NGS sequencing and data analysis should be performed to identify candidate biomarkers in complex diseases.

## Conclusion

In summary, we compared Accel, Accel PCR-free, ThruPLEX, NEXTFLEX and QIAseq WGS protocols for the analysis of variants from ccfDNA. The detection of germline variants, somatic SNVs, INDELs and CNVs were compared using control fragmented DNA samples and ccfDNA samples at 10X or 30X sequencing coverage.

Due to the lack of standard processing for ccfDNA, a workflow for sample preparation was performed to maximize the yield of ccfDNA including centrifugation, extraction, quantification, size analysis and normalization of samples. A process was established for library construction starting with 5–10 ng of input and for 10X or 30X sequencing: the number of cycles of the final PCR step of all samples are determined using qPCR assays, adapter primers are diluted for NEXTFLEX, and QIAseq methods and quantification using the same qPCR assay and library size analyses are performed to optimal pooling conditions.

Accel PCR-free did not provide enough product for sequencing. Accel, NEXTFLEX, ThruPLEX and QIAseq kits enabled to perform SNV, INDEL and CNV analyses starting with 10 ng (or 5 ng for NEXTFLEX) of ccfDNA. All kits do not have significant difference in percentage of PE reads and show similar results especially for SNVs and CNVs signatures. Although each kit detects unique variants, they have a significant common SNVs, INDELs and CNVs. There are more differences when detecting variants due to the various types of ccfDNA than due to the kits used. The variabilities between kits depend on type of samples, coverage used and type of variant. Accel detected more variants and specific SNVs, INDELs and CNVs compared to the other kits. ThruPLEX allowed the detection of more low-abundant somatic mutations than the other kits. Accel and NEXTFLEX signatures are alike and ThruPLEX and QIAseq have similar SNV signatures.

In the context of precision medicine, the identification of non-invasive candidate biomarkers could be performed using a standardized workflow including sample preparation, sequencing method and data analysis. Each step should be optimized using sensitive and accurate methods. The combination of common variants identified using the four kits could enable the analysis of disease specific pathways from ccfDNA. The variant patterns could be used to identify new subgroups of patients.

## Methods

### Samples

Reference for human genome sequencing (http://www.internationalgenome.org/data-portal/sample/NA12878), NA12878 DNA sample was purchased from CEPH (Paris, France). To mimic ccfDNA, 500 ng of DNA was fragmented using a Covaris E220 (Brighton, UK) with 10% of duty factor Peak Incident Power (W) 175, cycles per burst 200 during 320 s. Multiplex I cfDNA Reference Standard Set (HD780) and Structural Multiplex cfDNA Reference Standard (HD786) were also used for sequencing data analysis (Horizon Discovery, Waterbeach, UK). Multiplex I cfDNA Reference standard Set in Synthetic plasma from Horizon Discovery (HD786) was used to control the efficiency of the plasma extraction.

Commercial human plasma samples were purchased from BIOVIT (Burgess Hill, UK): breast 1 (female, stage IIA and 55 years), breast 2 (female, stage IIIA and 60 years), prostate (male and 51 years), colon (male, stage IIA and 68 years) from cancer patient, healthy individual 1 (female 50 years), healthy individual 2 (male of 60 years) and a pool of healthy male donors 3. The informed consent was obtained from all subjects. Plasma samples contained 1.5 to 1.8 mL, K2-EDTA and stored at −80 °C until their extraction.

Before the extraction step, plasma samples were centrifuged at 16,000 g during 10 min^33^. Then, ccfDNA from plasma was extracted using QIAamp Circulating Nucleic Acid Kit (Qiagen, Les Ulis, France) according to manufacturer’s instructions with 100 µL of elution volume.

Samples were quantified using the dsDNA HS Qubit Assay (Life Technologies, Illkirch, France) and fragment sizes were analysed using High Sensitivity DNA (Agilent Technologies, Les Ulis, France) chip on a 2100 Bioanalyzer Instrument. Samples were then concentrated at 1 ng/µL in water.

### Whole-genome sequencing

The ThruPLEX Plasma-seq Kit (Rubicon Genomics, Ann Arbor, USA), the QIAseq cfDNA All-in-One kit (Qiagen, Courtaboeuf, France), the NEXTFLEX Cell Free DNA-seq for Illumina kit (Biooscientific, Austin, USA), the Accel-NGS 2 S PCR FREE DNA Library for Illumina kit and the Accel-NGS 2 S PLUS DNA Library for Illumina kit (Swift Biosciences, Ann Arbor, USA) were used.

Experiments were performed according to the manufacturer’s protocol, starting with 10 ng of input except for the NEXTFLEX kit (5 ng for 10X or 10 ng for 30X) with the following optimizations.

To avoid adapter dimers, adapters were diluted at 70% for the QIAseq kit and 40% for the NEXTFLEX kit. Then, PCR libraries were purified by magnetic beads according to the manufacturer’s protocol for both ThruPLEX and NEXTFLEX kits and with the following modification ratio of 0.8X for QIAseq.^34^.

Library preparations were obtained using optimal number of cycles of library PCR for each sample. The determination of number of PCR cycles were performed on a LightCycler 480 thermocycler (Roche Applied Science, Penzberg, Germany) before the final PCR of the library preparation. Conditions for the QIAseq qPCR were 5 µL of 2X HIFI PCR Mix, 0.3 µL of Primers mix, 0.5 µL of 20X EvaGreen (Biotum, Fremont, USA) and 1.175 µL of ligation product in a 10 µL volume. The QIAseq cycling conditions included an initial denaturation step for 2 min at 98 °C, followed by 25 cycles of 20 s at 98 °C, 30 s at 60 °C, 30 s at 72 °C, followed by 1 min at 72 °C and a final HOLD at 4 °C. Conditions for the NEXTFLEX qPCR were 2.4 µL of NEXTFLEX PCR Master Mix, 0.4 µL of NEXTFLEX Primer mix, 0.5 µL of 20X EvaGreen and 0.9 µL of ligation product in a 10 µL volume. The NEXTFLEX cycling conditions included an initial denaturation step for 2 min at 98 °C, followed by 25 cycles of 30 s at 98 °C, 30 s at 65 °C, 1 min at 72 °C, followed by 4 min at 72 °C and a final HOLD at 4 °C. Conditions for the ThruPLEX qPCR were 1 µL of Indexing Reagent, 4.3 µL of Library Amplification Buffer, 0.2 µL of Library Amplification Enzyme, 0.5 µL of 20X EvaGreen and 1 µL of ligation product in a 10 µL volume. The ThruPLEX cycling conditions included an initial extension step for 3 min at 72 °C, then 2 min at 85 °C, followed by 2 min at 98 °C, followed by 4 cycles of 20 s at 98 °C, 20 s at 67 °C, 40 s at 72 °C, followed by 25 cycles of 20 s at 98 °C, 50 s at 72 °C and a final HOLD at 4 °C. Conditions for the Accel qPCR were 2 µL of Low EDTA TE, 1 µL of Reagent R1, 0.8 µL of Reagent R2, 2 µL of Buffer R3, 0.2 µL of Enzyme R4, 0.5 µL 20X EvaGreen and 1 µL of ligation product in a 10 µL volume. The Accel cycling conditions included an initial denaturation step for 30 s at 98 °C, followed by 25 cycles of 10 s at 98 °C, 30 s at 60 °C, 60 s at 68 °C, followed a final HOLD at 4 °C. The optimal number of PCR cycles without over-amplifying is determined using N-2 cycles of the qPCR assay for QIAseq, ThruPLEX and Accel and N-3 cycles of the qPCR assay for NEXTFLEX which N corresponds to half of the maximum fluorescent intensity of the qPCR assay.

Each library preparation was then quantified by qPCR assay in 10 µL volume using KAPA SYBR FAST Universal qPCR kit (Roche Applied Science) according to manufacturer’s protocol analyzing two dilution of 1:10,000 and 1:100,000 of each sample in triplicates. The size of the library was also determined using the Bioanalyzer Instrument.

Library preparation was carried out at 4 nM with 3% of PHIX (Illumina, San Diago, USA) in Elution Buffer (Qiagen) using equimolarity 4-plex per lane for 10X read depth or one sample per lane for 30X read depth (Illumina, San Diego, USA) on Illumina HiSeq X Series sequencer by 2 × 150 bp paired-end.

### Data treatment

FASTQ files were aligned on the human genome (GRCh37, version hs37d5 including decoys) using bwa software (version 0.7.15)^49^. Duplicate sequences were referenced and eliminated from the BAM files using Sambamba (version 0.6.8)^50^. An additional step of realignment was performed on the BAM files using GATK programs (RealignerTargetCreator and IndelRealigner)^29^. Coverage analyses have been generated using an in house pipeline based on metrics generated by Bedtools^51^ programs (version 2.17.1).

Identification of germline variants was performed using HaplotypeCaller from GATK version 4. Annotation of the VCF file was carried out using snpEff^52^ and snpSift^53^ based on data available in the Ensembl (GRCh37) and dbNSFP^54^ database (version 2.9).

Identification of somatic variants was performed using Mutect2 from GATK version 4, a somatic SNP and INDEL caller that combines the DREAM challenge-winning somatic genotyping engine of the original MuTect^30^ with the assembly-based machinery of HaplotypeCaller.

VCF files were filtered using VCFtools (0.1.12) for germline variants having a mapping quality ≥ 43 and a coverage ≥ 5 for 10X WGS and 13 for 30X samples^55^.

Copy number variation detection was performed by IchorCNA tool based on the Hidden Markov Model (HMM)^31^. A binning of 50 kb were used to detect both small targeted CNV and a 500 kb were performed to detect CNV of the other samples.

### Sample analysis

For the comparison of germline mutations of the NA12878 sample, TPR and PPV were calculated. TPR is defined by $TPR=\frac{TP}{TP+FN}$ and PPV is defined by $PPV=\frac{TP}{TP+FP}$where TP is the number of true positives, FN is the number of false negatives and FP is the number of false positives.

T-tests were performed to compare the percentage of PE between all kits. Hypergeometric tests were performed to test the significance of the overlap (under or over representation) between two kits for the detection of variants from the same plasma sample^56^. Bonferroni-corrected p-values have been computed.

Custom R scripts were used to compare samples and to perform venn diagram and PCA of the samples. Pathway enrichment analysis are performed using pathfindR R package^57^.

The MutationalPatterns R package was used to extract the mutational signatures of plasma samples^58^. VCF files of somatic mutation were used to construct a matrix with mutation counts. Then, mutational signatures were extracted from this mutation count matrix by non-negative matrix factorization (NMF) using optimized factorization rank of five^59^. CNV calling obtained by ichorCNA tool were used to build a matrix that contains the absolute copy number for each sample and segment. Then, CNV signatures were extracted from this matrix by NMF R package using optimized factorization rank of three^59^.

## Supplementary information

Supplementary information.
