# Supplementary material for: Comparison of commercially available whole-genome sequencing kits for variant detection in circulating cell-free DNA
Source: Sci Rep. 2020 Apr 10;10:6190. doi: 10.1038/s41598-020-63102-8 (PMC7148341; doi:10.1038/s41598-020-63102-8)
Supplement: Supplementary file 1 — Supplementary information. [file 41598_2020_63102_MOESM1_ESM.pdf]

Comparison of commercially available whole-genome sequencing kits for variant detection in circulating cell-free DNA

Florence Mauger, Caroline Horgues, Morgane Pierre-Jean, Nouara Oussada, Lilia Mesrob and Jean-François Deleuze.

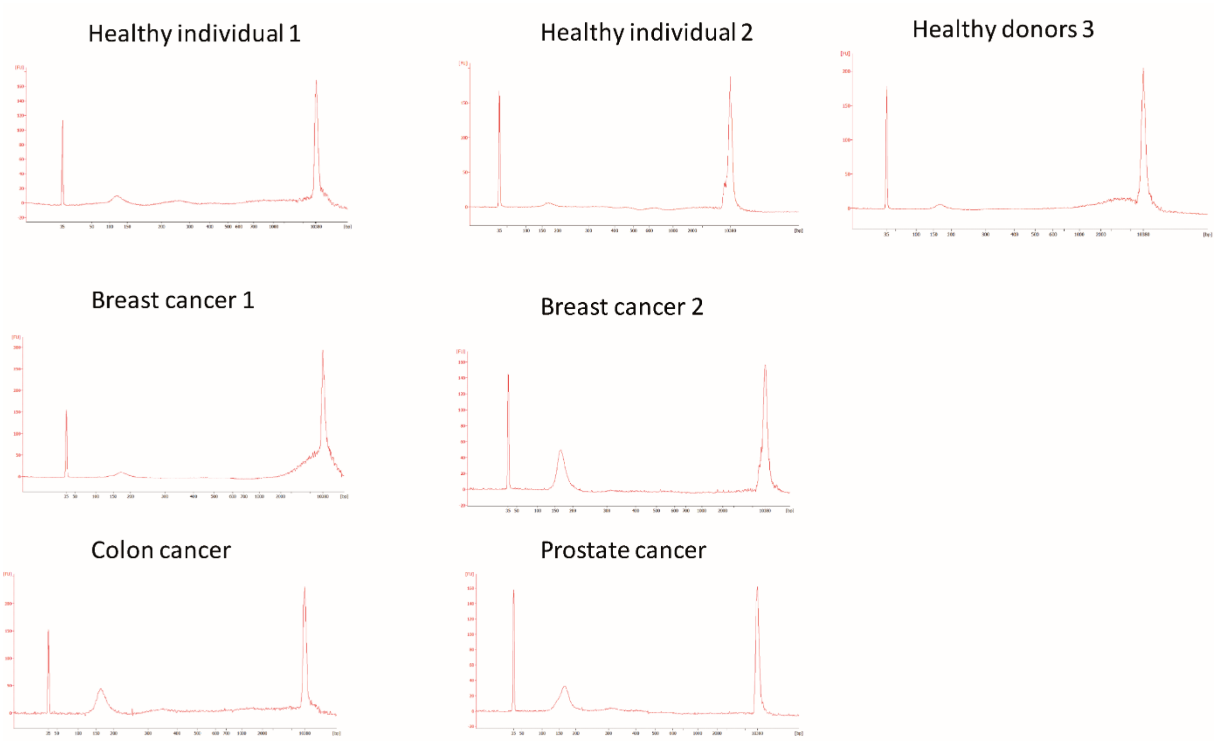

Figure S1: Fragment size analysis of ccfDNA extracted from healthy individual 1, healthy individual 2 and healthy donors 3, prostate cancer, colon cancer, breast cancer 1, breast cancer 2 plasma.

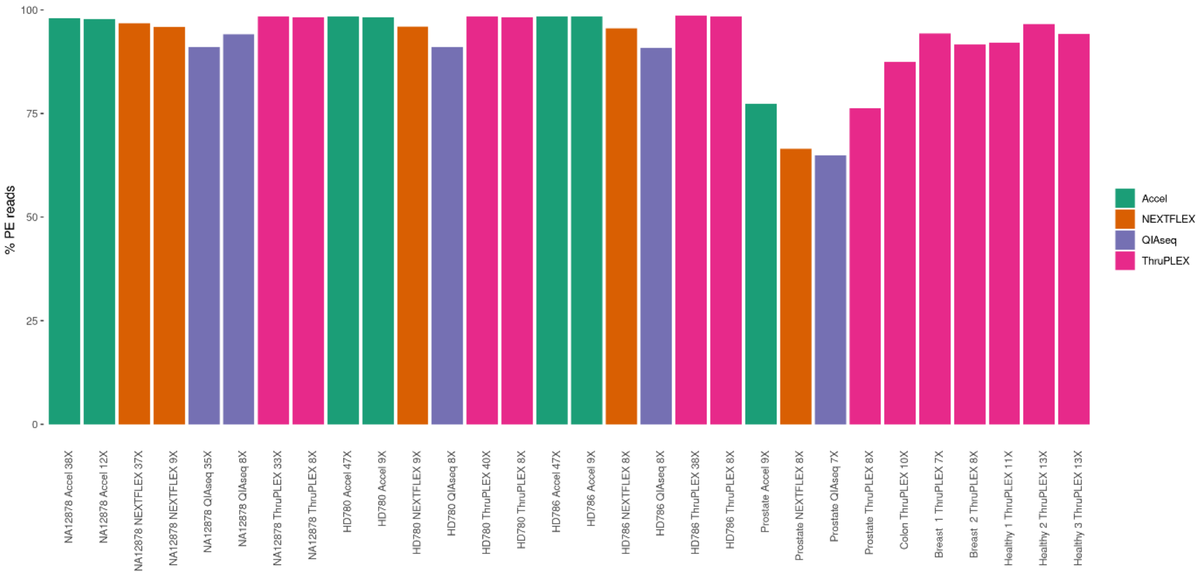

Fig.S2: median coverage and percentage of PE reads of WGS of Accel in green, NEXTFLEX in red, QIAseq in purple and ThruPLEX in pink.

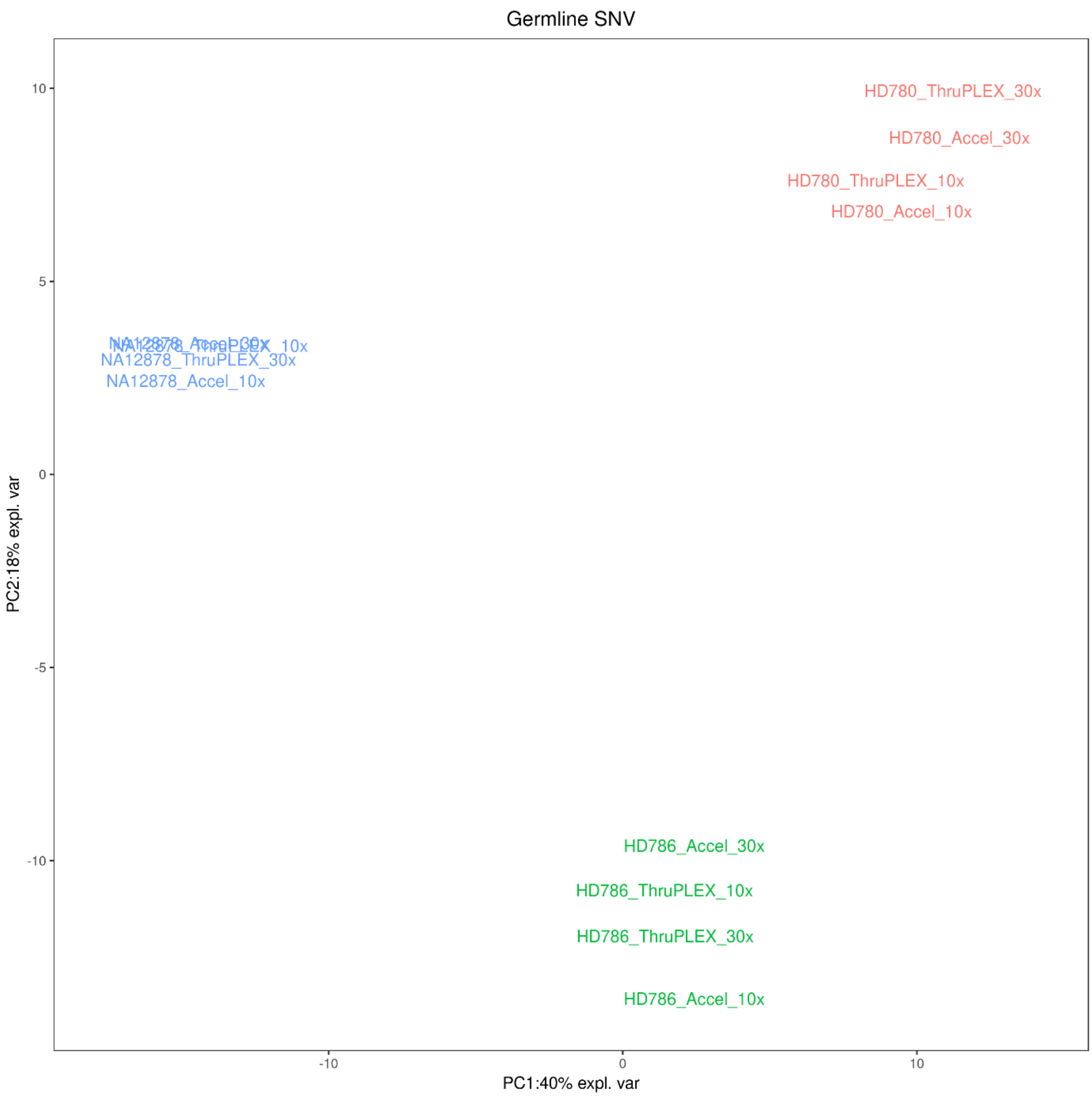

Figure S3: Principal component analysis of germline SNV for Accel, and ThruPLEX WGS of NA12878, HD780 and HD786 samples at 10X and 30X. NA12878 sample is blue, HD786 sample is green, and HD780 sample is red.

KEGG ID	Pathway	Count	p-value	Gene
hsa03040	Spliceosome	10	1.7e-16	SNRPD2, SNRPD3, SNRNP70, SF3A1, SF3A2, SF3A3, PHF5A, CHERP, LSM4, LSM7, SNRNP40, PQBP1, CCDC12, THOC2, RBMX, RBMXL3, SRSF4
hsa04120	Ubiquitin mediated proteolysis	10	7.5e-13	UBA1, SAE1, UBE2A, UBE2G2, UBE2NL, UBE3A, HUWE1, HERC2, PPIL2, XIAP, BIRC8, PIAS4, AIRE, MID1, RBX1, KLHL13, CUL4B, FZR1
hsa04064	NF-kappa B signaling pathway	10	1.5e-09	LCK, BTK, CARD10, TICAM1, CD40LG, TNFSF14, TAB1, TAB3, TNFRSF13C, PIAS4, XIAP, CCL4, RELB, CCL13, GADD45B
hsa03010	Ribosome	10	4.9e-08	MRPS15, RPS4X, RPS4Y2, RPS11, MRPL34, RPL3, RPL13A, RPL18, RPL18A, RPL36, RPL39, UBA52, RPL36A
hsa04961	Endocrine and other factor-regulated calcium reabsorption	10	6.7e-06	PRKACB, AP2A1, AP2B1, AP2S1, CLTCL1, ATP1B4, KLK2, KLK1, PRKCG
hsa04919	Thyroid hormone signaling pathway	10	7.1e-06	PRKACB, MAP2K2, MAPK1, HDAC1, EP300, MED12, MED14, MED1, NOTCH3, PRKCG, SLC9A1, ATP1B4, SLC16A2, PIK3R2
hsa04810	Regulation of actin cytoskeleton	10	8.1e-06	LPAR4, FGF21, PDGFB, ITGAE, ITGB2, FGD1, RRAS, ARHGEF6, PIK3R2, ARAF, MOS, MAP2K2, MAPK1, ARHGAP35, RAC2, PAK3, DIAPH2, SLC9A1, PIP5K1C, LIMK2, WAS, WASF2, MSN, TMSB4X, TMSB4Y, MYH9
hsa05140	Leishmaniasis	10	2.9e-05	TAB1, ITGB2, NCF4, CYBB, MAPK1, ELK1, MAPK11, MAPK12, MARCKSL1
hsa03013	RNA transport	10	7.8e-05	NUP50, NUP62, RANGAP1, SUMO3, GEMIN8, EIF3I, EIF3D, EIF1AY, FMR1, THOC2, THOC5, NXF3, NXT2, UPF1, UPF3B
hsa04110	Cell cycle	10	9.5e-05	HDAC1, RBX1, CDC45, CCNB3, YWHAH, SMC1A, SMC1B, STAG2, FZR1, CHEK2, EP300, GADD45B, MCM5

Table S1: Pathway analysis of prostate cancer plasma individual using common genes of Accel, ThruPLEX, NEXTFLEX and QIAseq WGS.
